# Physical Restraints and Seclusion in Psychiatric Settings in the Eastern Mediterranean Region: A Systematic Review of the Perspectives of Nurses and Individuals with Mental Illness

**DOI:** 10.3390/healthcare14091161

**Published:** 2026-04-26

**Authors:** Asrar Salem Almutairi, Owen Price, Abdullah Hassan Alqahtani, Antonia Marsden, Karina Lovell

**Affiliations:** 1 Division of Nursing, Midwifery and Social Work, University of Manchester, Manchester M13 9PL, UK; owen.price@manchester.ac.uk (O.P.); karina.lovell@manchester.ac.uk (K.L.); 2 College of Nursing, Princess Nourah Bint Abdulrahman University, Riyadh 84428, Saudi Arabia; 3 Psychiatry Division, Medicine Department, Johns Hopkins Aramco Healthcare (JHAH), Dhahran 31311, Saudi Arabia; dr.alqahtani16@gmail.com; 4 Division of Population Health, Health Services Research & Primary Care, University of Manchester, Manchester M13 9PL, UK; antonia.marsden@manchester.ac.uk

**Keywords:** attitudes, perceptions, experiences, mental health nurses, individuals with mental illness, physical restraints, mechanical restraints, seclusion, eastern mediterranean region

## Abstract

**Background/Objectives**: Physical restraints and seclusion remain ethically contested interventions in psychiatric care, raising significant concerns regarding patient safety, dignity, and therapeutic impact. Despite growing international momentum towards restraint-reduction strategies, their use persists across the Eastern Mediterranean Region (EMR), an area that has been the subject of limited systematic attention. This review synthesises evidence on the knowledge, attitudes, and experiences of nurses and individuals with mental illness regarding these practices in EMR psychiatric settings. **Methods**: Following PRISMA 2020 guidelines (PROSPERO: CRD42023383751), we systematically searched nine electronic databases for studies published up to June 2023, supplemented by backward and forward citation searching. Multiple reviewers independently screened records against predefined eligibility criteria, with disagreements resolved through consensus. Methodological quality was assessed using Joanna Briggs Institute (JBI) Critical Appraisal tools, and reporting quality was evaluated using an adapted CROSS checklist; these two appraisal dimensions were conducted and reported independently. Findings were integrated through narrative synthesis. **Results**: From 4634 identified records, 19 studies conducted across 11 EMR countries met the inclusion criteria. Nursing knowledge deficits were identified across multiple settings, and attitudes towards restraint practices were predominantly negative. Individuals with mental illness consistently described restraint as humiliating, punitive, and physically distressing. Recurrent challenges identified across studies included inadequate staff training, chronic understaffing, and limited access to restraint-reduction alternatives. **Conclusions**: Substantial gaps in nursing knowledge and training persist across the EMR. The findings of this review, while derived predominantly from cross-sectional studies with convenience samples, suggest that evidence-based education programmes, standardised restraint-reduction policies, and patient-centred care frameworks warrant prioritisation to safeguard the rights, safety, and dignity of individuals with mental illness in this region. Longitudinal and experimental research is needed to confirm these directions and establish their effectiveness within EMR contexts.

## 1. Introduction

Physical restraint (PR), mechanical restraint (MR), and seclusion, hereafter referred to as restraint and seclusion (R/S), are coercive measures used in psychiatric care to control acute behavioural disturbances and prevent harm to patients and/or others [1]. PR refers to direct or device-assisted physical holding used to limit a patient’s freedom of movement, while MR involves the use of physical devices (e.g., belts and limb ties) to restrict mobility, and seclusion is the involuntary confinement of a patient in isolation in a designated room under continuous observation [2]. Despite being intended to ensure the safety of the patient and others, these methods have been under debate and subject to criticism because of their potential negative effects on patients’ physical and mental well-being [2]. Despite the criticism, in some situations, mental healthcare professionals, such as nurses, may need to employ these methods as a last resort, e.g., when other strategies have failed or there is an imminent risk to the patient or others [3]. R/S is typically used after evaluating a patient’s behaviour, risk levels, and the availability of less restrictive alternatives [1]. According to Larue et al. [4], in psychiatric care, R/S interventions are a risk-reduction and safety measure. However, ethical, legal, and moral implications should be considered when these interventions are used [5]. Möhler and Meyer [5] have stated that the potential negative consequences of restraint and seclusion, such as physical injury, decreased self-esteem, psychological distress, and deterioration of the therapeutic relationship, are debated among clinicians and critics. On the other hand, proponents argue that judicious use of R/S within a structured care model may prevent serious harm and promote a safe environment [6].

The ethical rationale for using R/S is based on the conflict between the need to respect patient autonomy and the clinical need to avoid causing harm [5]. International human rights organisations and clinical guidelines increasingly recommend eliminating or reducing the use of coercive measures in mental healthcare, given the potential for re-traumatisation and erosion of therapeutic relationships [1]. Systematic reviews of nurses’ knowledge and practices regarding the use of physical restraint, which have mainly been conducted in geriatric and general medical settings, have revealed knowledge deficits among nurses, inconsistencies in practices, and a lack of standardisation of training on restraint use [5]. Studies conducted on psychiatric inpatient care have also shown that staff attitudes, institutional culture, and the availability of training programmes influence the extent and appropriateness of the use of R/S [3].

The Eastern Mediterranean Region (EMR) is a challenging context for mental healthcare because of the presence of country-wise barriers and similarities [7]. The EMR countries share many cultural, social, and economic characteristics that affect the practice of mental healthcare. In addition, there is a social stigma against seeking mental healthcare in this region [8]. Mental healthcare delivery in the EMR is subject to many challenges. Most countries in the region suffer from a lack of services and inequity in access to care due to inadequate human resources, infrastructure, and financial resources [9,10]. Furthermore, there is a significant lack of specialised mental healthcare in rural areas, limiting access to appropriate and timely care [10]. Cultural beliefs and practices have a significant influence on help-seeking behaviours and treatment choices. Countries in the EMR also share similar characteristics regarding political instability, conflict, and displacement, all of which can affect the population’s mental health and well-being [7]. All these contextual and structural determinants are expected to impact the prevalence and nature of coercive measures in psychiatric care; however, this issue has hardly been explored. The World Health Organization’s Comprehensive Mental Health Action Plan 2013 to 2030 calls for the progressive elimination of coercive practices and the promotion of a human-rights-based approaches to mental healthcare across the globe [11]. Therefore, exploring R/S in understudied regions such as the EMR is essential. Nurses are a vital part of the multidisciplinary team in the healthcare system, particularly in the mental health sector, where their role transcends basic care, involving the application of various therapeutic interventions and the management of challenging behaviours, such as aggression. Nurses’ understanding and perception of the application of R/S affect the quality of care they provide to their patients [3]. Nurses should have adequate knowledge of the indications and contraindications for such interventions and monitoring and should account for legal and ethical considerations in order to make appropriate decisions [5]. Similarly, the perceptions of individuals with mental illness are essential; many patients who have been subjected to coercive measures describe them as humiliating, punitive, and psychologically harmful, and their voices must be heard to reform patient-centred policies [1,4]. Thus far, several single-country studies conducted within EMR countries have explored the perspectives of nursing staff regarding R/S. However, no systematic reviews have attempted to synthesise the evidence comprehensively at the regional level; likewise, no reviews have assessed the perspectives of both nursing staff and individuals with mental illness in the EMR context. This systematic review is designed to bridge this knowledge gap by synthesising the available evidence on the knowledge, attitudes, and experiences of both nurses and individuals with mental illness regarding the use of PR and S in psychiatric care across the Eastern Mediterranean Region. Hopefully, the findings of this review will contribute to the development of evidence-based training modules, contextually sensitive clinical protocols, and policies for reducing the use of restraint.

## 2. Materials and Methods

This systematic review was conducted according to the Preferred Reporting Items for Systematic Reviews and Meta-Analyses (PRISMA) 2020 standard [12], which was followed to ensure comprehensive and transparent reporting. The PRISMA checklists pertaining to this investigation have been incorporated into the Supplementary Materials (Tables S1 and S2).

### 2.1. Eligibility Criteria

We employed a mixed-methods systematic review design, integrating both quantitative (cross-sectional, observational, and other non-randomised designs) and qualitative (interview-based) study designs. We selected this approach to gain a comprehensive understanding of nurses’ knowledge, attitudes, and experiences (KAE), recognising that both numerical data and contextual perspectives are necessary to capture the complexity of restraint and seclusion practices in psychiatric settings.

The inclusion of studies in this review was contingent upon meeting the following eligibility criteria. Studies were included if they

1. Examined the knowledge, attitudes, and/or experiences of nurses or individuals with mental illness regarding the use of physical and/or mechanical restraints and seclusion within mental health settings in the Eastern Mediterranean Region;
2. Employed quantitative methods (cross-sectional, observational, or other non-randomised designs), qualitative methods (interviews, case studies), or mixed methods;
3. Were conducted in inpatient mental health or psychiatric facilities;
4. Included participants aged 18 years or older;
5. Were published in English or Arabic;
6. Were peer-reviewed primary research articles, with no restrictions imposed on publication date.

Exclusion Criteria

Studies were excluded if they

1. Were published in languages other than English or Arabic;
2. Were conducted outside the Eastern Mediterranean Region;
3. Did not focus on mental health or psychiatric settings;
4. Did not include nurses as participants or focused solely on other healthcare professionals without reporting nurse-specific data;
5. Did not address knowledge, attitudes, or experiences regarding restraint, seclusion, or coercive measures;
6. Were editorials, commentaries, opinion pieces, or literature reviews rather than primary research;
7. Focused exclusively on paediatric or child/adolescent psychiatric populations;
8. Focused on the mechanical or pharmacological aspects of restraint without addressing staff or patient perspectives;
9. Were duplicate publications (the most recent or complete version was retained);
10. Lacked sufficient methodological detail or data from which relevant information could be extracted.

Only published peer-reviewed articles were included in this review. Grey literature (unpublished studies, theses, conference abstracts, and grey reports) was not systematically searched.

### 2.2. Protocol Development

The protocol for this systematic review was prospectively registered with PROSPERO prior to data extraction (registration number, CRD42023383751; year registered, 2023). The detailed protocol can be accessed at https://www.crd.york.ac.uk/PROSPERO/view/CRD42023383751 (accessed on 22 April 2026).

### 2.3. Search Strategy

A systematic search was conducted across nine electronic databases to identify relevant studies. The following databases were searched: CINAHL, MEDLINE (Ovid), EMBASE, PubMed, ASSIA, Web of Science, Google Scholar, PsycINFO, and PsycARTICLES.

The search strategy combined three concept blocks using Boolean operators.

Block 1 (Population):

(‘nurse*’ OR ‘psychiatric nurs*’ OR ‘mental health nurs*’ OR ‘healthcare professional*’ OR ‘patient*’ OR ‘individual* with mental illness’).

Block 2 (Intervention):

(‘physical restraint*’ OR ‘mechanical restraint*’ OR ‘seclusion’ OR ‘coercive measure*’ OR ‘containment’ OR ‘isolation’ OR ‘chemical restraint*’).

Block 3 (Outcomes):

(‘knowledge’ OR ‘attitude*’ OR ‘perception*’ OR ‘experience*’ OR ‘practice*’ OR ‘view*’ OR ‘perspective*’).

The following search filters were applied: English or Arabic language; human studies; no date restrictions. The final search date was 30 June 2023.

Database-specific search strings (which include Boolean operators, controlled vocabulary terms and filters) are provided in Supplementary File S1; the two parts of each search were Search 1 (staff perspective), which included the population block, staff block, restraint block, cognition block and geographical block, and Search 2 (patient perspective), which was identical except for using the patient block in place of the staff block.

### 2.4. Study Selection

The results of the database search were exported to Covidence (Veritas Health Innovation, Melbourne, Australia, https://www.covidence.org/, accessed in 2023) and examined for duplicate removal. The following phased study selection process was applied.

Phase 1: Title and Abstract Screening

The lead researcher (A.S.A.) independently screened all titles and abstracts. To ensure comprehensive coverage and methodological rigour, all records were systematically distributed among three supervisors (O.P., A.M., and K.L.) and one psychiatry expert (A.H.A.), with each reviewer independently assessing their assigned portion. Any disagreements were resolved through discussion until consensus was achieved.

Phase 2: Full-Text Assessment

Two reviewers (A.S.A. and A.H.A.) independently assessed the full texts of all potentially eligible studies against the inclusion criteria. Disagreements were resolved by consensus. O.P. and K.L were consulted as a third reviewer when agreement could not be reached.

The inclusion criteria encompassed primary research publications that specifically examined the knowledge, attitudes, and experiences of mental health nurses and individuals with mental illness regarding the use of physical/mechanical restraints and seclusion in mental health facilities within the Eastern Mediterranean Region.

Additional Identification Methods: Backward and Forward Citation Searching

**Figure 1:**
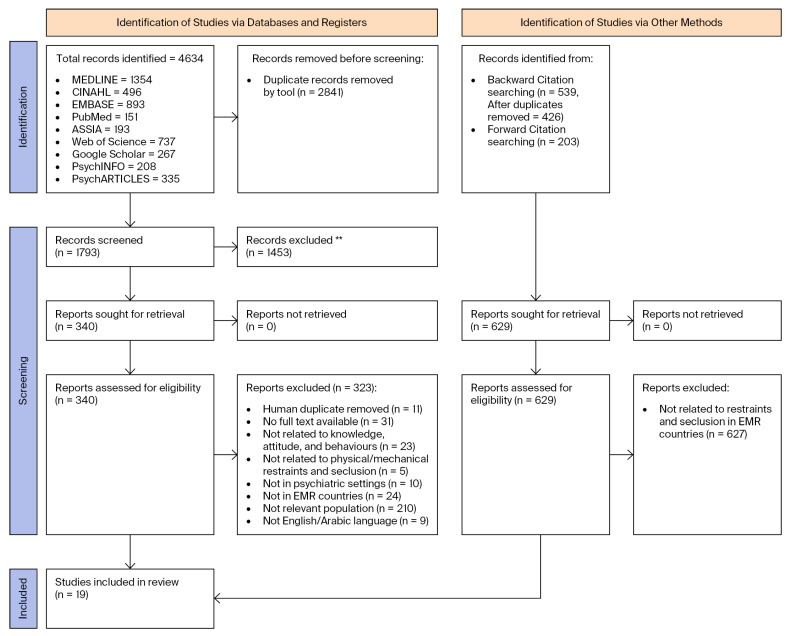
PRISMA 2020 flow diagram for the systematic review. ** Duplicate records were removed using automation tools (*n* = 2841), while records excluded (*n* = 1453) were excluded during title and abstract screening by reviewers.

To supplement the electronic database searches, backward and forward citation searching was conducted for all 19 studies meeting the final inclusion criteria. Backward citation searching involved reviewing the reference lists of each included article; forward citation searching was carried out via Google Scholar and Web of Science to identify studies subsequently citing each included article. The number of citations reviewed per study is documented in Supplementary File S2. No additional eligible studies were identified through either procedure. Both methods are reflected in the PRISMA 2020 flow diagram (Figure 1) under References from other sources.

### 2.5. Review Process

Phase 3: Data Extraction

Two reviewers (A.S.A. and A.H.A.) independently extracted data using the Covidence (Veritas Health Innovation, Melbourne, Australia; https://www.covidence.org/, accessed in 2023). Covidence was used for title/abstract screening, full-text review, and data extraction throughout the review process. A data collection tool developed by the Joanna Briggs Institute (JBI) was used to establish a systematic data collection framework, ensuring consistency and comprehensiveness in data collection. The form included key sections relevant to the review objectives, including participant information, study methods, interventions, and outcomes.

Data Extraction Framework

For each study included, the following data were extracted across three outcome domains:

- Domain 1—Knowledge, comprising the knowledge assessment tools and scales used; mean scores and standard deviations; specific knowledge gaps identified; and factors associated with knowledge levels.
- Domain 2—Attitudes, encompassing attitude measurement instruments; proportions of positive versus negative attitudes; attitudes towards restraint, seclusion, and alternatives; and associations with demographic characteristics or clinical experience.
- Domain 3—Experiences, encompassing qualitative themes and narratives; practice behaviours (e.g., monitoring frequency, frequency of restraint use); patient responses and outcomes; and barriers to and facilitators of practice.

Handling Domain Overlap: When data addressed multiple domains, information was coded to the primary domain based on the study’s stated focus and additionally noted in secondary domains as applicable.

Pilot Testing: A pilot extraction was conducted on three randomly selected studies (representing approximately 15% of the included studies) by both reviewers independently prior to full extraction. Minor refinements were made to the extraction form based on the pilot findings to improve clarity and consistency.

Phase 4: Adjudication

All discrepancies in extraction were resolved through discussion between the two reviewers (A.S.A. and A.H.A.), with O.P, K.L and A.M. providing a final decision when consensus was not achieved.

The two independent reviewers (A.S.A. and A.H.A.) conducted structured discussions to clarify any discrepancies or ambiguities regarding study eligibility. The systematic review process, including searching, selection, and data collection, was conducted between June and November 2023.

### 2.6. Quality Appraisal

To assess the quality of the included articles, two reviewers (A.S.A. and A.H.A.) applied the following Joanna Briggs Institute (JBI) [13] critical appraisal tools according to each study’s design.

JBI checklists applied according to study design:

- Cross-sectional studies: JBI Checklist for Analytical Cross-Sectional Studies (8 items);
- Qualitative studies: JBI Checklist for Qualitative Research (10 items);
- Observational studies: JBI Checklist for Prevalence Studies (9 items).

Quality-rating system:

Each criterion was scored as Yes = 1/No = 0/Unclear = 0. Quality categories were defined as follows:

- High quality—≥75% of items were scored as ‘Yes’.
- Moderate quality—50–74% of items were scored as ‘Yes’.
- Low quality—<50% of items were scored as ‘Yes’.

The JBI tools provided a systematic, evidence-based approach that encompassed key methodological elements, including study design, sampling strategies, data collection methods, and data analysis procedures, facilitating assessment of overall methodological rigour, reliability, and internal validity.

The CROSS checklist [14] was used in addition to the JBI tools to assess the completeness and transparency of reporting of the included studies. Originally developed for survey research, the CROSS checklist was adapted for the range of non-survey study designs included in this review. The JBI critical appraisal tools and the adapted CROSS checklist were applied as two independent and complementary appraisal dimensions: the former assessed methodological quality encompassing study design, sampling, data collection, and analytical procedures while the latter evaluated the completeness and transparency of reporting across all included studies.

A full account of all adaptations applied to the CROSS checklist, specifying which items were retained, reworded, or omitted for non-survey study designs, is provided in Supplementary File S3.

Discrepancies in quality appraisals between reviewers were resolved through discussion; O.P. and K.L were consulted as a third reviewer when consensus could not be reached. The results of the quality appraisals provided a comprehensive overview of the strengths and limitations of each included study. All included studies were summarised in narrative form and presented in a summary table to facilitate clear and transparent communication of findings.

### 2.7. Data Synthesis

Data synthesis was carried out by integrating the quantitative and qualitative findings from the included studies, following a convergent parallel design.

Data were synthesised following the narrative synthesis framework proposed by Popay et al. [15], proceeding through the following four stages:

- Preliminary Synthesis—summary tables were developed for each outcome domain (Knowledge, Attitudes, and Experiences), capturing study characteristics and key findings to enable systematic comparison across studies.
- Exploration of Relationships—patterns were examined across studies according to geographic location (EMR countries), study design (quantitative vs. qualitative), participant type (nurses vs. individuals with mental illness), and outcome domain (K, A, E).
- Thematic Analysis—for qualitative data and findings from interview-based studies, thematic analysis was conducted through initial independent coding by two reviewers (A.S.A. and A.H.A.), followed by grouping of codes into categories and overarching themes, which were refined iteratively until consensus was reached.
- Integration—quantitative findings (e.g., proportions reporting negative attitudes) were used to contextualise qualitative themes (e.g., reasons underlying negative attitudes), thereby providing a more complete and coherent picture of the evidence.

#### Software and Tools

Citation screening and data extraction were managed using Covidence (Veritas Health Innovation, Melbourne, Australia; https://www.covidence.org/, accessed in 2023). Thematic analysis of qualitative findings was conducted manually by two independent reviewers (A.S.A. and A.H.A.), following Popay et al.’s framework for narrative synthesis [15]. Emerging themes were discussed iteratively until a consensus was reached.

## 3. Results

### 3.1. Study Characteristics

Inter-reviewer agreement was assessed using Cohen’s Kappa coefficient (κ) at each stage of the review process. Agreement was strong at the title and abstract screening stage (κ = 0.82) and for data extraction (κ = 0.80), and it was substantial at the full-text review stage (κ = 0.78) and for quality appraisal (κ = 0.76). All values exceeded the accepted threshold of κ ≥ 0.60 for systematic reviews, indicating there was substantial to strong agreement across all review stages.

A total of 4634 records were identified through the database search, with the following distribution across databases: MEDLINE (1354); CINAHL (496); EMBASE (893); PubMed (151); ASSIA (193); Web of Science (737); Google Scholar (267); PsycINFO (208); and PsycARTICLES (335) (see Figure 1). After the review process, 19 studies were included in the review, while 340 reports were sought for retrieval. Of the reports excluded (*n* = 323), the reasons for exclusion included duplicate removal (*n* = 11); no full text being available (*n* = 31); not being related to knowledge, attitudes, or experiences (*n* = 23); not being related to physical/mechanical restraints and seclusion (R/S) (*n* = 5); not having been conducted in psychiatric settings (*n* = 10); not having been conducted in Eastern Mediterranean Region (EMR) countries (*n* = 24); not focusing on a relevant population (*n* = 210); and not being in English or Arabic (*n* = 9).

The characteristics of the included studies are summarised in Table 1. The studies covered various aspects, including knowledge (9 studies), attitudes (12 studies), and experiences (10 studies). In terms of methodology, most of the studies used a questionnaire or survey design (15 studies), while one study was conducted using in-depth unstructured interviews, one was conducted using semi-structured interviews, one was conducted using an observational checklist, and one was conducted using an assessment-structured interview schedule. The participants were primarily psychiatric nurses (18 studies) and individuals with mental illness (3 studies). The number of participants varied: 8 studies had fewer than 50 participants, 11 studies had between 50 and 150 participants, and 1 study had more than 150 participants. The studies were conducted across 11 countries within the EMR: Saudi Arabia (4 studies), Egypt (4 studies), Tunisia (3 studies), Iran (2 studies), Iraq (1 study), Sudan (1 study), Kuwait (1 study), the United Arab Emirates (1 study), Palestine (1 study), Jordan (1 study), and Pakistan (1 study).

### 3.2. Findings from Domain 1: Mental Health Nurses

The Findings for This Domain Are Summarised in Table 2.

#### 3.2.1. Knowledge

International evidence highlights that in-service education programmes have a significant positive impact on psychiatric nurses’ knowledge, attitudes, and practices regarding the use of restraints [35]. Within the EMR context, knowledge of R/S has been found to be acceptable or satisfactory in some studies and inadequate in others. For example, Aslam et al. [18] and Thomas [21] found generally acceptable or satisfactory levels of knowledge among psychiatric nurses in Pakistan and Saudi Arabia, respectively, whereas Abdel-Hussein and Mohamed [25] found that the knowledge of psychiatric nurses in Iraq regarding R/S was generally inadequate. Differences in education, training, and healthcare systems may contribute to the differences in knowledge regarding R/S.

Aslam et al. [18] found that the overall level of R/S knowledge among psychiatric nurses in Pakistan was satisfactory and not significantly related to the socio-demographic variables pertaining to the participants. These authors argued that factors such as the availability of regular in-service training, hospitals’ policies and guidelines, and cultural values may have contributed to the participants’ satisfactory levels of knowledge. Thomas [21] found that psychiatric nurses in Saudi Arabia had acceptable or satisfactory levels of knowledge about R/S. Specifically, the participants had high levels of knowledge pertaining to the correct indications and monitoring strategies for R/S.

In contrast, Abdel-Hussein and Mohamed [25] found that psychiatric nurses in Iraq had inadequate knowledge about R/S, particularly regarding the basic concepts. The authors argued that the inadequate levels of knowledge among the participants may be attributed to the lack of adequate coverage of the subject matter in their educational programmes, inadequate training, and the lack of a protocol for R/S. Taken together, it could be said that, although some psychiatric nurses have acceptable or satisfactory levels of knowledge about R/S, knowledge gaps still exist, and standardised training and continuous education on the subject matter and clear policies and guidelines about the use of R/S should be provided to ensure it is applied safely.

#### 3.2.2. Attitude

A total of 12 studies discussed nurses’ attitudes towards R/S, revealing generally negative or neutral attitudes in EMR countries. The differences in attitude may be attributed to differences in staffing levels, the availability of legal protection, and education.

The most negative attitudes towards R/S were found in a study by Ahmed et al. [24] conducted in Egypt, with 78% of the sample having a negative attitude. The authors also found a significant positive association between knowledge, attitudes, and practices (*p* < 0.05). Another study by Morsi and Sabra [32] also investigated Egyptian nurses’ attitudes towards R/S, but the results revealed less negative attitudes. Aslam et al. [18] did not find significant associations between nurses’ attitudes and sociodemographic characteristics (*p* > 0.05).

Legal protection is one of the common reasons for using R/S. Three studies conducted in Saudi Arabia by Thomas [21], Hasan and Abulattifah [27], and Khalil et al. [28] found that a significant percentage of the sample of nurses perceived that the use of R/S was a way of protecting them legally even though they admitted that there are alternatives to R/S. In Palestine, R/S was perceived as effective but sometimes overused [30].

A number of studies found that attitudes towards R/S were associated with the perception of inadequate staffing levels. In the UAE [19], Saudi Arabia [27], and Sudan [29], nurses frequently link R/S use to insufficient staff. A Sudanese study found a significant association between attitudes and practices (*p* < 0.05). In addition, in Kuwait, researchers found differences in nurses’ attitudes towards R/S based on gender and years of experience [34]. Al-Maraira et al. [26] found that a structured education significantly improved nurses’ attitudes towards R/S (*p* < 0.001).

Overall, the results of the review revealed that nurses’ attitudes towards R/S are generally neutral or negative. The findings suggest that attitudes may be more influenced by contextual rather than sociodemographic factors. Improving knowledge through standardised and evidence-based education may have a positive influence on attitudes.

#### 3.2.3. Experiences

This discussion on factors that influenced psychiatric nurses’ use of R/S in the EMR cannot be concluded without referring to contextual factors. Among these factors are staff shortages and a lack of options. Thomas [21] found that most of the Saudi sample used R/S as a last resort. Similarly, in Iraq, Abdel-Hussein and Mohamed [25] found that staff shortages and a lack of other interventions were the most frequent reasons nurses provided to justify the use of R/S. From these findings, one could conclude that staff shortages and a lack of other interventions might lead to more frequent use of R/S in any setting.

The emotional responses of nurses who used R/S varied. For example, in Saudi Arabia, Hasan and Abulattifah [27] found that less than half of their sample of psychiatric nurses felt guilty or ashamed for using R/S, potentially indicating a lack of integration of ethical principles into practice. Mahmoud [29] found that nearly 40% of the sample of psychiatric nurses felt guilty after using R/S and that there was a significant relationship between attitudes towards R/S and its actual use, possibly indicating that ethical principles have been integrated into practice and that nurses are aware of patients’ feelings. In Palestine, Al-Awawdeh et al. [30] found that nurses sometimes used R/S more often than needed, which might indicate an awareness of this tendency without taking any actions to avoid it.

Generally, the experiences of the psychiatric nurses who participated in the selected studies indicate that the use of R/S was not a matter of individual decision-making or judgement; rather, it was influenced by contextual factors, particularly staff shortages, a lack of other options, and the prevailing organisational culture. Moreover, the fact that emotional responses were not reported in all studies indicates that ethical awareness alone might not lead to changes in practices.

##### Analytical Synthesis

When considered collectively, the evidence suggests that nurses’ experiences of R/S use across EMR settings reflect a pattern of institutional constraint rather than individual clinical preferences. The recurring reports of staffing shortages and an absence of alternatives as being primary drivers indicate that R/S may represent a systemic accommodation to resource limitations. The tension between nurses’ professional values reflected in attempts to exhaust alternatives prior to R/S and the organisational pressures that nonetheless precipitate its use underscores the need for interventions that target structural and organisational determinants alongside individual training and attitudinal development. Strategies aimed at reducing R/S should therefore address ward-level staffing adequacy, access to evidence-based de-escalation alternatives, and the development of institutional cultures that support reflective practices and ethical accountability.

### 3.3. Findings from Domain 2: Individuals with Mental Illness

The findings for this domain are summarised in Table 3.

#### 3.3.1. Knowledge

Three studies examined the knowledge of individuals with mental illness regarding R/S, drawing on both formal and experiential sources of understanding. Yahyavi and Shahvari [16] conducted a hermeneutic phenomenological study with 20 hospitalised patients in Tehran, Iran, using in-depth unstructured interviews. The analysis identified four overarching themes and 12 sub-themes that collectively characterise patients’ lived understanding of R/S: captivity (limitation and suffocation); abuse (humiliation, violation, and harassment); oppression (innocence, sadness, hatred, and fear); and punishment (waiting for freedom for reflection and waiting for recovery) [16]. These themes suggest that patients develop a rich and layered conceptual understanding of R/S that is rooted in its lived psychological impact rather than its clinical rationale.

Kamel et al. [33] explored the understanding of 80 psychotic inpatients at a psychiatric hospital in Alexandria, Egypt, using a descriptive exploratory design. The patients’ accounts regarding the reasons for R/S use were largely congruent with those provided by nursing staff, suggesting that patients possessed a degree of situational awareness regarding the clinical contexts in which R/S was applied. When asked about perceived alternatives to R/S, 65% of the patients preferred ‘giving time and care’, 13.75% identified medication as an alternative, and 21.25% believed that there are no alternatives to R/S [33]. These preferences indicate that a substantial proportion of patients perceive de-escalation and relational care as viable alternatives, while a significant minority may have internalised R/S as an inevitable practice.

Elgamal [34] included 30 inpatients from a psychiatric hospital in Kuwait. A formal knowledge assessment was not conducted in this study; however, the findings revealed that 86% of patients reported feelings of helplessness, and 90% experienced mixed and confusing emotions following R/S [34], suggesting that even in the absence of structured knowledge measurement, patients demonstrated an awareness of the emotional and psychological consequences of R/S that shaped their understanding of the practice.

##### Analytical Synthesis

Across these three studies, the evidence indicates that individuals with mental illness acquire a form of situated knowledge regarding R/S through direct lived experience rather than through formal education or clinical instruction. This experiential knowledge, encompassing awareness of triggers, emotional sequelae, and perceived alternatives, is qualitatively distinct from the procedural knowledge assessed in nursing staff studies. Importantly, the findings reported by Yahyavi and Shahvari [16] and Elgamal [34] suggest that the depth of patients’ conceptual understanding of R/S does not attenuate the practice’s perceived punitive character; the acquisition of experiential knowledge appears to coexist with, rather than mitigate, the psychological harm associated with R/S. This distinction underscores the importance of centring patient perspectives in clinical decision-making and policy development, recognising that comprehension of a practice’s rationale does not equate to acceptance of its implementation.

#### 3.3.2. Attitudes

The attitudes of individuals with mental illness towards R/S were examined in two studies, both of which revealed predominantly negative orientations characterised by perceived loss of autonomy, dignity, and therapeutic trust. Kamel et al. [33] found that the majority of patients at an Alexandria psychiatric hospital reported negative feelings towards R/S, including humiliation and worthlessness (38.75%) and anger and bitterness (25%). These findings suggest that R/S is perceived by patients as a degrading experience that diminishes their dignity and sense of self-worth [33].

Similarly, Elgamal [34] reported that R/S was not accepted by 46% of patients in Kuwait, while 86% reported feelings of helplessness and 63% considered the experience to be a form of punishment. The attitudes described in both studies indicate that patients perceive R/S as a punitive rather than therapeutic measure, a perception reinforced by the stigmatising nature of physical restriction, the associated feelings of isolation, and the profound sense of lost autonomy and control [33,34].

The negative attitudes identified across these studies may reflect several interconnected factors, including the stigmatising nature of R/S as a visible marker of behavioural dyscontrol, the sense of isolation imposed by its application, and patients’ perceptions of diminished agency over their own care. These attitudinal patterns are consistent with international evidence documenting the erosion of therapeutic relationships following coercive interventions.

##### Analytical Synthesis

The findings from both studies collectively indicate that there is a fundamental gap between the clinical framing of R/S as a safety-oriented therapeutic intervention and the subjective experience of individuals with mental illness, for whom it appears to be associated with dehumanisation, punishment, and a loss of personhood. This gap between the stated therapeutic intent of R/S and its experienced reality as punitive and dignity-violating suggests that the values underpinning R/S in EMR psychiatric settings may not be adequately communicated to or shared with the individuals subjected to this practice. Addressing this gap would require not only the development of patient-centred communication strategies but also a broader institutional commitment to meaningful patient involvement in care planning and restraint-reduction initiatives.

#### 3.3.3. Experiences

The experiences of individuals with mental illness regarding R/S, as documented across three EMR studies, were consistently characterised by psychological distress, physical pain, and a profound sense of dehumanisation. Kamel et al. [33] reported that the most common emotional responses experienced by patients during R/S use in Alexandria, Egypt, were worthlessness and humiliation (38.75%), followed by anger and bitterness (25%), while only 3.75% reported that R/S had a calming effect. Physical reactions to R/S were also prevalent, with 85% of patients reporting physical symptoms, including generalised body aches (100% of those affected) and severe pain in the extremities (4.4%) [33]. These findings suggest that feelings of humiliation and anger may be associated with the negative emotional impact of R/S and that the physical sequelae of restraint may contribute to its overall distressing character.

Elgamal [34] demonstrated that 90% of patients in Kuwait report conflicting and confusing emotional responses following R/S use, suggesting that the experience of R/S is frequently overwhelming and emotionally disorienting. A substantial proportion of patients (46%) did not consider R/S appropriate, 86% felt powerless during its application, and 63% reported feeling that they were being punished [34]. These findings indicate that for many individuals with mental illness, R/S may be perceived as a punitive rather than therapeutic measure, suggesting that the clinical intent of R/S is frequently not communicated to or experienced by patients in a way that is consistent with its stated therapeutic rationale.

The phenomenological dimensions of the R/S experience were most comprehensively documented by Yahyavi and Shahvari [16], whose hermeneutic phenomenological study involving 20 inpatients in Tehran identified four overarching thematic categories—captivity, abuse, oppression, and punishment—that encapsulate the existential and psychological dimensions of the R/S encounter. These themes collectively suggest that R/S is experienced not merely as a clinical procedure but as a fundamental violation of personhood, evoking responses that are consistent with the experience of institutional trauma [16].

Restraint and seclusion (R/S) should be applied only as a last resort when there is an imminent risk of harm to a patient individual or others, with priority given to preserving the dignity and well-being of individuals with mental illness. Healthcare professionals should strive to minimise the adverse impact of R/S by ensuring that less restrictive alternatives are consistently explored, that patients are informed of the reasons for any intervention, and that their recovery needs are prioritised throughout and following the episode of R/S.

##### Analytical Synthesis

Across all three studies conducted in Iran [16], Egypt [33], and Kuwait [34], a consistent cross-country pattern in which individuals with mental illness perceive R/S as criminalising, dehumanising, and physically painful emerges. The recurrence of themes such as punishment, helplessness, humiliation, and loss of autonomy across culturally and geographically distinct EMR settings suggests that these are not context-specific phenomena but rather structural features of how R/S is experienced by those subjected to it. These findings are consistent with international evidence documenting the traumatogenic potential of coercive practices in psychiatric care, including systematic reviews conducted in Western European and North American contexts. The evidence from these EMR studies further supports the development and implementation of trauma-informed care frameworks, which centre the psychological safety, dignity, and autonomy of individuals with mental illness and feature active patient involvement in care decisions. Restraint-reduction strategies informed by such frameworks, including the development of crisis intervention protocols, investment in de-escalation training, and the co-production of safety plans with patients, are strongly indicated by the collective weight of the patient-reported experiences documented in these studies.

### 3.4. Quality of Reporting

The reporting quality of the 19 studies included was assessed with the adapted version of the CROSS checklist [14], measuring the completeness and transparency of study reporting rather than methodological quality (study design and conduct), which will be discussed in the following section (Section 3.5).

Of the 19 studies, three achieved high reporting quality, clearly stating outcome measures, data collection, and population descriptions (Aslam et al. [18]; Thomas [21]; Hasan and Abulattifah [27]). Other studies did not present a sampling frame for recruitment, thereby limiting the assessment of potential selection bias and the extent of generalisability to other settings (Aloulou et al. [23]; Maatouk et al. [20]; El Kefi et al. [22]). Two papers did not report ethical approval (Kamel et al. [33]; Elgamal [34]), which is a concern because the populations involved in all studies included vulnerable groups, such as those living with psychiatric conditions. Dawood [31] and Moghadam et al. [17] also provided an incomplete description of the data analysis processes, limiting the extent to which the methods could be replicated and the trustworthiness of findings assessed.

The 2 qualitative studies provided sufficient details to contextualise findings and the reflexivity of the research team (Yahyavi and Shahvari [16], *n* = 20; Moghadam et al. [17], *n* = 14) to ensure moderate reporting quality, although both papers provided insufficient details regarding the data analysis to be able to assess the transparency of the identification of themes (Yahyavi and Shahvari, [16], *n* = 20; Moghadam et al., [17], *n* = 14).

In summary, overall reporting quality of the 19 studies included in this review was mixed. The main problems identified included the lack of a sampling frame, lack of ethical approval reporting, and incomplete description of data analysis. Despite the limitations identified, the included studies represent useful contributions to the existing evidence base and future studies should aim to provide more information on these areas to improve the transparency of reporting for more robust future syntheses. See Supplementary File S3 for further reporting criteria assessment.

### 3.5. Methodological Quality of the Included Studies

The methodological quality appraisal results for the design, sampling, data collection, and analysis of individual studies are presented in this section. It should be noted that this process is separate from the evaluation of reporting quality presented in Section 3.4 above. Each quality appraisal criterion was answered as Yes = 1, No = 0, or Unclear = 0. The studies were then categorised as high (≥75% “Yes”), moderate (50–74% “Yes”) or low quality (<50% “Yes”) in line with the approach outlined in Section 2.6. Differences were discussed until consensus was reached, and if necessary, a third reviewer was involved.

The JBI [13] Quality Assessment Checklist was used as follows: Analytical Cross-Sectional Studies (8 items) are 13 studies, Qualitative Research (10 items) are 2 studies, Prevalence Studies (9 items) are 4 studies (Table 4). The results indicate that 11 studies were of high quality, 6 were of moderate quality and 2 were of low quality.

Aslam et al. [18], Abdulla et al. [19] and Thomas [21] were considered to be of high quality as most JBI criteria were met. These studies were well-designed, reporting the inclusion criteria, the sampling methods and the process of data collection in sufficient detail to allow their internal validity to be evaluated, although the design is cross-sectional. Maatouk et al. [20] used an appropriate design but was limited by convenience sampling and a small sample size, reducing generalisability. Aloulou et al. [23] and Moghadam et al. [17] were primiarily considered low quality, as they did not provide enough information in their reports about the process of sampling, the data collected and the process of analysis, which is necessary to evaluate the internal validity of the study. The results of Ahmed et al. [24] and Abdel-Hussein and Mohamed [25] provide important quantitative evidence, but the lack of any triangulation with qualitative research limits the depth of interpretation of the quantitative results.

Several studies, including Hasan and Abulattifah [27] and Khalil et al. [28], did not report potential sources of bias or conflicts of interest. However, all studies reported evidence of cultural sensitivity. The research by Yahyavi and Shahvari [16] and Kamel et al. [33] contributed valuable qualitative data, but neither reported on the ethical considerations or the characteristics of the sample to any extent in their reports. While some of the studies were of good quality, most were constrained by convenience sampling, small sample size and incomplete reporting of data analysis. We considered these findings, which are distinct from the reporting findings, during the discussion of our interpretation of the findings.

### 3.6. Influence of Quality Appraisal on Data Interpretation

The results of the quality appraisal had a significant impact on the analysis of the synthesised findings. Methodologically robust, well-reported studies (Aslam et al. [18]; Thomas [21]) contributed to the narrative synthesis primarily in terms of mapping the body of evidence regarding knowledge gaps and the mainly negative attitudes towards R/S in EMR contexts. Studies that had low or moderate methodological quality or were poorly reported (Aloulou et al. [23], Moghadam et al. [17]) were interpreted with caution and contributed to the analysis in a supplementary rather than central manner, mainly being used to contextualise findings from higher-quality studies.

The methodological quality assessment (using JBI tools [13]) and the quality of reporting assessment (using an adapted version of the Cross Checklist [14]) were done separately and in different sections (Section 3.4 and Section 3.5). The findings from these complementary assessments were used to inform how each study was weighted in the narrative synthesis of the studies.

The findings are consistent with those of other systematic reviews, which report a diverse range of methodological approaches and inconsistent reporting standards in psychiatric nursing research conducted in low- and middle-income countries in the EMR and which, like the present study, highlight the need for research-capacity-building in the region. As such, studies of low or moderate quality were interpreted with caution and, where possible, contributed to the analysis in a supplementary rather than central manner.

## 4. Discussion

This systematic review has summarised the available evidence from 19 studies, conducted in 11 EMR countries, on the knowledge, attitudes, and experiences of nurses and patients regarding the use of R/S in psychiatric settings. The three major themes emerging from the findings of the included studies are (1) the diversity of the knowledge of psychiatric nurses, (2) the predominately negative attitudes of nurses towards R/S, and (3) the consistently negative—and sometimes traumatic—experiences of patients who have been subjected to R/S. The three themes are discussed below in the context of the international literature, and their implications for clinical practice and policy are highlighted.

### 4.1. Nursing Knowledge

Psychiatric nurses’ knowledge of R/S varied significantly from one EMR country to another. This variation could be due to differences in the availability and accessibility of educational resources for nurses rather than a lack of capability of individual nurses. Studies conducted in Pakistan [18] and Saudi Arabia [21] report generally acceptable levels of knowledge, whereas research in Iraq [25] and Tunisia [23] shows significant deficits in legal requirements, monitoring protocols, and de-escalation alternatives. Other knowledge deficits reported in two or more studies include lack of understanding of (1) medical orders [27,28], (2) monitoring every two hours [21,27], (3) patient’s rights and ethics [25,28], and de-escalation techniques [18,24,31]. These knowledge deficits are consistent with the results of studies conducted in Western [5] and Asian [36] countries. Such consistency indicates that nurses’ educational preparation for managing disturbed behaviours is inadequate worldwide. Within the EMR, the wide variability in the knowledge of nurses indicates differences in the healthcare systems, the educational preparation of nurses, and the development and implementation of policies relevant to R/S across EMR countries. Therefore, these findings suggest that the development of regional frameworks for nursing education on R/S may represent a productive avenue for practice improvement, though the effectiveness of such frameworks in the EMR context remains to be evaluated empirically.

### 4.2. Nurses’ Attitudes

Nurses’ attitudes towards R/S were predominately negative [24,29,31] or ambivalent. For example, a study conducted in Egypt reported that 78% of nurses held negative attitudes towards R/S [24], while a study conducted in Sudan reported that nurses had ambivalent attitudes [29]. It appears that the negative or ambivalent attitudes of nurses towards R/S are not due to the attributes of individual nurses but are instead linked to moral and ethical concerns about the use of coercive measures [29] and recognition of their harmful effects [31]. In three studies [19,27,29], a shortage of staff was identified as one of the factors necessitating the use of R/S in the psychiatric ward. This result indicates that improving nurses’ attitudes alone may not necessarily lead to a reduction in the use of R/S unless accompanied by improvements in the structural factors, such as improving the staff-to-patient ratio. A study conducted in Egypt found a positive relationship between nurses’ knowledge and attitudes towards R/S [24]. Such findings are consistent with the results of studies conducted in other parts of the world [36]. However, being cross-sectional, these studies do not provide evidence of the effect of knowledge on attitudes. Accordingly, the association between knowledge and attitudes observed across EMR studies should be interpreted as hypothesis-generating rather than confirmatory, and longitudinal or experimental designs are required before any causal relationship can be assumed.

An experimental study conducted in Jordan found that nurses’ attitudes towards R/S improved following an educational intervention [26]; however, determining the generalisability of such findings to the context of other EMR countries requires further research. In general, the negative attitudes towards the use of R/S appear to be a universal attribute of mental health nurses [3,37,38].

### 4.3. Experiences of Individuals with Mental Illness

The experiences of patients subjected to R/S were explored in three studies [16,33,34], which were conducted in Iran [16], Egypt [33], and Kuwait [34]. Overall, patients described their experiences of R/S as dehumanising [16], punitive [34], and physically painful [33]. Patients reported feelings of captivity [16], humiliation [16], oppression [16], and helplessness [34]. Such experiences are congruent with the international literature [1,4], indicating that the use of coercive measures, such as R/S, is often perceived as re-traumatising and that the use of such measures should be minimised [11]. Although the use of coercive measures, including R/S, to prevent patients from harming themselves or others may be justified [2,6], these procedures must be implemented in a way that respects patients’ dignity and rights and promotes therapeutic relationships between patients and mental health staff.

### 4.4. Implications for Practice

The findings of this review, while derived from a methodologically heterogeneous evidence base in which cross-sectional designs predominate and causal inference is not possible, nonetheless identify consistent and recurrent patterns that carry meaningful implications for clinical practice and health policy across the EMR. These implications should be understood as evidence-informed directions for practice development rather than definitive prescriptions. Future longitudinal research and experimental testing are needed to confirm findings within the regional context.

Across multiple jurisdictions R/S educational knowledge deficits were consistently noted related to legal frameworks [23], physician orders [27,28], patient monitoring [21,27], ethical and patient rights [25,28] and de-escalation techniques [18,24,31]. These findings in multiple sites represent an urgent need for structured evidence-based R/S education as a component of nursing workforce development, and an indication of a broad-based systemic gap in contemporary training programmes. Contextually relevant R/S education needs to be incorporated into all pre-registration programmes [26] and ongoing professional/project development, although optimal design and delivery of such education require further investigation.

Staffing shortages were identified multiple times as a significant driver of R/S usage [27,29,31], pointing out the role of structural constraints in effecting change that may be greater than individual level influences. Therefore, educational strategies alone are not sufficient to accomplish change without complementary organisational reform that targets staffing, resource allocation and organisational culture. The relative impacts of structural and individual level determinants, however, are still to be clearly defined, thereby requiring longitudinal and mixed methods research.

In so far as recurrent defects in procedural compliance related to documentation [30], patient oversight [21] and review of restraint episodes [31] suggest the necessity for a standardised protocol covering the entire restraint/seclusion pathway from the initiation of restraint/seclusion to the evaluation of the restraint/seclusion episode following the event. Whether these deficiencies reflect the absence of an appropriate policy or the lack of effective implementation of existing policies is uncertain; thus, there is the need for governance and audit-related research within Electronic Medical Record (EMR) systems.

While the research is scant and methodologically limited, all the patient-based research [16,33,34] found that patients perceived restraint/seclusion to be dehumanising, punitive and distressing. Furthermore, the findings are consistent with international literature [1,4]. Collectively, this research supports the inclusion of the patient within the care planning process (including the development of advanced directives, crisis plans and safety agreements) as part of restraint reduction strategies. Some research has also identified that improving de-escalation skills [26] and offering additional non-coercive alternatives to restraint/seclusion [21,26,30,33] may reduce the incidence of coercive restraint; however, the effect sizes relating to EMR are unknown. Collectively, these implications are consistent with the international evidence recommending the use of sensory-based care, relational de-escalation and structured post-incident review as non-coercive alternatives to restraint/seclusion [6,11].

### 4.5. Strengths

The strengths of this review include its focus on studies conducted in a specific region (the EMR), synthesis of quantitative and qualitative evidence, use of systematic and bilingual literature searches, and use of dual-reviewer screening with high inter-rater agreement (Cohen’s Kappa ranged between 0.76 and 0.82). Such strengths enhance the relevance and validity of the findings and conclusions.

### 4.6. Limitations

A few limitations are important when interpreting results from this review. There are many cross-sectional studies and no causal inference can be made due to these cross-sectional designs. The prevalence of using convenience samples limits how well these studies can be generalised to all psychiatric nursing in all areas of the EMR. Three of the studies considered opinions of patients; therefore, experience from patients with mental illnesses is not well represented in studies conducted within the EMR, and this is an area that should be given priority in any future study. Differences in method between studies (variances in outcome measures and analysis) did not allow for a quantitative meta-analysis to be performed and required a narrative synthesis to be performed which has a greater chance of subjective interpretations by the researchers performing the review. The search strategy excluded literature not published within the region that was grey or published in a non-English or non-Arabic language; therefore, evidence from studies that are outside of the EMR may have been excluded from this review which added to potential publication bias. The search was conducted until June 2023, and therefore studies published after the study will not have been included. Thus, any updates to this review should occur as additional evidence becomes available in the coming years.

### 4.7. Comparison with Non-EMR Evidence

The three major themes identified in this review, i.e., the diversity of nurses’ knowledge, nurses’ negative attitudes, and patients’ negative and traumatic experiences, are consistent with the results of studies conducted on psychiatric nurses in other parts of the world [3,37,38]. Although the underlying causes of such attitudes and experiences may differ due to differences in the structure and function of healthcare services and socio-cultural factors between the EMR and other parts of the world, comparative studies would be valuable in order to explore the similarities and differences between various parts of the world. Doing so could help reveal effective strategies for reducing the use of R/S in various contexts.

## 5. Conclusions

This systematic review of 19 studies from 11 EMR countries found consistent evidence of knowledge gaps amongst psychiatric nurses about legal and procedural aspects of R/S; predominantly negative attitudes related to inadequate staffing levels and training; and a common perception amongst people with mental illness that R/S is degrading, penalising, and psychologically traumatic. These findings suggest that well-designed educational initiatives for nurses on legal frameworks, monitoring procedures, and alternatives to R/S consistent with the WHO Comprehensive Mental Health Action Plan 2013–2030 [11] and NICE guidelines on the management of violence and aggression [6] warrant prioritisation across the region. The evidence further underscores the importance of person-centred care approaches that meaningfully involve individuals with mental illness in decision-making regarding their own care.

Longitudinal and interventional studies assessing the impact of education on the use of R/S in psychiatric care in the EMR are required. Country-specific research must be conducted in EMR countries where no evidence was found, including Syria, Lebanon, Yemen, and Libya. Research co-produced with people with mental illness is also necessary to ensure that the perspectives of individuals with mental illness will influence policy changes.

## Figures and Tables

**Table 1:** Characteristics of the studies.

Characteristics	Number of Studies
KAE studies ^1^:	
Knowledge	9
Attitude	12
Experience	10
Method ^2^:	
Questionnaire/survey	15
In-depth unstructured interview	1
Semi-structured interview	1
Observational checklist	1
Assessment-structured interview schedule	1
Participants ^2^:	
Psychiatric nurses	18
Individuals with mental illness	3
Number of participants ^2^:	
<50	8
50–150	11
>150	1
Country ^2^:	
Saudi Arabia	4
Egypt	4
Iraq	1
Iran ^3^	2
Sudan	1
Tunisia	3
Kuwait	1
United Arab Emirates	1
Palestine	1
Jordan	1
Pakistan	1

^1^ A single study could assess all components (knowledge, attitude, and experience). ^2^ A single study could cover different participants, methods, and countries. ^3^ Two studies were conducted in Iran [16,17].

**Table 2:** Studies about psychiatric nurses’ KAE regarding R/S.

Reference	Study Design	Participants and Setting	Sampling	Methods	Main Findings		
					Knowledge	Attitudes	Experiences
Aslam et al. [18]	Descriptive cross-sectional study design	150 Psychiatric nurses in Lahore, Pakistan.	Non-probability convenience sampling	Questionnaire	Knowledge was insignificantly associated with sociodemographic and other factors (*p*-value > 0.05). Mean ± SD of the levels of knowledge of PR scale was 7.01 ± 0.89.	Attitudes were insignificantly associated with sociodemographic and other factors (*p*-value > 0.05). Mean ± SD of the levels of attitude of PR scale was 35.04 ± 3.	Practices were found to be significantly associated with gender, marital status, the type of special course attended, and previous training acquired (*p* < 0.05). Mean ± SD of the levels of practice PR scale: 36.26 ± 1.
Abdulla et al. [19]	Exploratory cross-sectional design	307 (response rate: 89.6%) healthcare professionals (54.18% nurses) from three healthcare facilities treating mental illness in Abu Dhabi and Dubai, T UAE	Convenience non-sampling method	Questionnaire survey	NA	88% said they apply coercive measures only when necessary. 84.4% said it is necessary to restrain patients mechanically. 56.8% said they use MRs to manage violence if there are not enough staff members.	NA
Maatouk et al. [20]	Descriptive cross-sectional design	30 nurses from the psychiatry department of Razi hospital in Manouba City, Tunisia.	NR	Questionnaire	23.30% of nurses underwent mental health training at the beginning of their professional careers. 50% of nurses underwent training focused on PR.	NA	83.3% used PR for psychomotor agitation. 56.6% ignored the psychological effects of the PR on patients. 73.3% informed patients before restraining them.
Thomas [21]	Descriptive design	138 nurses (response rate: 86%) working in inpatient non-critical areas in Tabuk, Saudi Arabia.	Convenience	Self-administered questionnaire	A sufficiently high level of knowledge about use of restraints was reported (the mean score was above average: 26.31 ± 2.60). 66.4% reported that restraints should be released every 2 h if the patient is awake. 34.4% said that restraints should be used when one cannot watch a patient closely. 82.4% said that patients are allowed to refuse restraint. 64.8% reported that use of PR requires a family member to sign a consent form. 52.8% reported that good alternatives to restraint do not exist.	Participants had a favourable attitude towards the use of PR (the mean score was 30.57 ± 4.56). 48.8% believe that family members have the right to refuse the use of restraints. 48% disagreed with the notion that patients lose their dignity when placed in restraints. 36.6% felt guilty about using restraints. 12.8% reported that the main reason for using restraints is being short-staffed. 48.8% reported that applying restraints assures legal protection of patients and hospital staff.	99.2% reported trying alternative nursing measures before resorting to restraint. The majority of nurses collaborate to discover alternative ways of controlling patients’ behaviour as opposed to using restraints.
El Kefi et al. [22]	Descriptive design	25 healthcare professionals (14 nurses) from the psychiatry department of The Principal Military Hospital of Instruction of Tunis (HMPIT), Tunisia.	NR	Self-administered questionnaire	NA	NA	52% used seclusion. 72% had no specific training. 12% felt that no accidents could occur in seclusion. 20% said that it is unnecessary to transcribe the monitoring parameters on the medical file.
Aloulou et al. [23]	Descriptive correlation, cross-sectional	30 psychiatric nurses from the psychiatric department of the general hospital in Sfax, Tunisia.	NR	Questionnaire	Insufficient knowledge of the practice of PR was reported. 90% have not received training in PR. 26.6% consider it an emergency measure. 73.3% believe no medical prescription is required for PR. 40% reported that PR is the last therapeutic option. 80% reported that PR cannot be employed as a punitive measure. 86.7% felt that PR should not be applied because of a lack of nursing staff. 53.3% reported that PR should be avoided for people over 65 years old.	NA	NA
Ahmed et al. [24]	Exploratory descriptive research design	100 nurses from psychiatric emergency departments in Cairo, Egypt.	Convenience	A structured questionnaire + observational checklist	42% had a satisfactory level of knowledge regarding PR.	78% had negative attitudes toward PR. 22% had positive attitudes toward PR. There was a significant positive correlation between practices and knowledge and attitude as well as between knowledge and attitude (*p*-value < 0.05).	75% did not explain the reason for employing the intervention to the patient. 59% did not monitor skin and circulation. 32% reviewed the physician’s orders for application. 36% recorded use of PR.
Abdel-Hussein & Mohamed [25]	Descriptive correlation, cross-sectional	41 psychiatric nurses from 2 psychiatric teaching hospitals in Baghdad, Iraq.	Purposive	Questionnaire	Lack of knowledge on How to use R/S; Reasons for using R/S; Rules and regulations on the use of R/S; Alternatives to R/S; Complications pertaining to the use of R/S.	NA	NA
Al-Maraira et al. [26]	Experimental study, repeated-measures time-series design with two-equivalent groups	48 psychiatric nurses from the national centre for mental health in Jordan.	Convenience (sample randomisation was applied)	Self-reported structured questionnaires; Staff Attitude Toward Coercion Scale (T1 = pretest, T2 = post-test immediately after training, T3 = post-test 2 weeks after training, and T4 = post-test 4 weeks after training).	NA	81% of the variance in attitudes was related to the intervention conditions. Overall attitude scores reduced significantly, by 12.54, between T1 and T4 (*p* < 0.001). The intervention group (M = 39.2) had significantly lower attitude scores than the comparison group (M = 57.0). There were significantly lower attitude scores at T2, T3, and T4. There were significant improvements in the intervention group’s mean attitude scores. There were group differences in attitudes towards coercive Measures Subscales scores over the 4 measurement points.	NA
Hasan & Abulattifah [27]	Descriptive explanatory cross-sectional design	110 nurses (response rate: 89%) from Al-Amal mental health government hospital in Jeddah, a large city in the KSA.	Convenient purposive sampling	Self-administered structured questionnaire.	88% reported that a specific physician’s order is required in order to administer PR. 88.1% reported that PR is associated with complications such as skin breakdown. 79% reported that restraints should be removed every 2 h. 60.9% believe that PR can be used without a physician’s orders. 69% reported that there are no sufficient alternatives to PR. 67% reported that confusion and disorientation are good reasons for using PR. 56.4% reported that nurses can use PR when there is no one else to monitor or control a patient.	The majority of the sample did not try to employ any other nursing measures before using restraints. Two-thirds of the sample cited staff shortages as the main reason why they use restraints. Less than half of the sample felt guilty using restraints or embarrassed when family members entered the room of the restrained patient. 46.9% applied restraints to assure legal protection for nurses and the hospital.	The majority of nurses do not assess the patient’s condition every 10 to 15 min (64.6%). 38.1% of nurses monitor skin status after using PR. 57.3% do not document the patient intervention. The majority of nurses do not talk to the patient while restraining them (80%). 76.4% of the nurses do not include the patient in the decision to use PR.
Khalil et al. [28]	A descriptive correlative exploratory design	37 nurses from an inpatient psychiatric hospital in Jeddah, Saudi Arabia.	Non-probability convenience sampling	Self-administered structured questionnaire.	The majority of nurses use R/S to protect patients or others (97.2%). The majority of nurses said that R/S should be ordered by a physician (88.9%). 22.2% said that garments should be strictly applied around the patient’s body. 27.8% reported that it is the patient’s right to refuse R/S.	33.3% prefer using both restraints and seclusion 82.9% reported that the hospital can apply R/S for legal protection. 80% believe that it is important to inform patients that R/S is used as a part of their care. 58.8% reported having no feelings of guilt when using R/S. Being male was correlated with the use of PR (r = −0.341). Use of seclusion had a positive significant correlation with nurses’ levels of education (r = 0.465).	The majority check on restrained or secluded patients at least every two hours (85.7%). 80.6% frequently assess if the restraints should be removed once a patient’s has condition improved. 42.9% of the participants disagreed with the notion that more patients need to be restrained or secluded if there is shortage of staff.
Mahmoud [29]	Descriptive research design	96 nurses from three mental hospitals and two mental wards in two general hospitals in Khartoum, Sudan.	Convenient purposive sampling	Self-administered structured questionnaire	NA	Most of the patients reported that the main reason restraints are used in the hospital is a shortage of staff (58.3%). 39.6% reported experiencing feelings of guilt. 61.4% reported trying to use an alternative. There was a significant association between total attitude score and practice regarding the use of PR. There was no association between attitude and practice in relation to nurses’ sex, education, and years of experience and workplace.	The majority reported that there are insufficient staff during PR (65.6%) 56.3% do not assess patient condition every 10–15 min. 33.3% do not involve the patient in making decisions.
Al-Awawdeh et al. [30]	Cross-sectional study	67 nurses from six wards in a mental health hospital in Palestine.	Convenience	Questionnaire	NA	Seclusion is one of the most effective approaches to managing violent patients (*p* < 0.05). Patients who are violent are restrained for their own safety (*p* < 0.05). PR is sometimes used more than necessary (*p* < 0.05). De-escalation is an effective way of preventing violence (*p* = 0.03).	NA
Moghadam et al. [17]	Qualitative research	14 nurses working in psychiatric hospitals in Ahvaz, Southern Iran.	Purposive	Semi-structured interviews	NA	NA	Four categories emerged: (1) Restraint as a multi-purpose procedure; (2) Processing of PR; (3) Restraint as a challenging subject; (4) The effects of restraint on the spectrum.
Dawood [31]	Descriptive correlation, Cross-sectional	128 psychiatric nurses (65 Egyptians and 63 Saudis) from three psychiatric hospitals (two Egyptian mental health hospitals in the central delta and one Saudi mental health hospital in the central region)	Convenience	Questionnaire survey	NA	64.06% reported that a restrictive environment can contribute to aggression. 67.18% reported that seclusion is most effective for violent patients. 92.18% reported that violent patients are restrained for their own safety. PR (53.12%)/seclusion (52.34%) is sometimes used more than necessary.	NA
Morsi & Sabra [32]	Descriptive design	50 nurses from Tanta mental health Hospital in Tanta, Egypt.	Convenience	Questionnaire	Restraint should be used when one cannot observe a patient closely (50%). Confusion or disorientation is the main reason for using a restraint (74%). In an emergency, a nurse can legally restrain a patient without a physician’s order (34%). Good alternatives to restraints do not exist (54%).	52% reported having a positive attitude toward using PR. Family (12%) and patients (12%) have the right to refuse to use restraint. 12% and 18% said that they feel guilty and embarrassed, respectively, when a patient is in restraints. 88% reported feeling knowledgeable about caring for restrained patients.	NA
Kamel et al. [33]	Descriptive exploratory design	108 nurses from a psychiatric hospital in Alexandria, Egypt.	NR	Assessment, structured interview schedule	NA	NA	95.4% use restraints for patient-oriented reasons (excitement, suicide attempts, aggressive behaviour, etc.). 43.5% use restraints for organisation-oriented reasons (trouble making, attempts to escape, etc.). 0.9% use restraints for behavioural therapy.
Elgamal [34]	Descriptive correlation, Cross-sectional	62 nurses from Kuwait state psychological medicine hospital (4 acute wards were included: 2 male wards and 2 female wards).	NR	Two questionnaires	NA	Male nurses prefer continuous restraint. Senior nurses prefer short periods of restraint. Patients who roam and are hyperactive can be restrained. Patients can be restrained upon their own request.	NA

NR = not reported; NA = not applicable; R/S = restraint/seclusion; PR = physical restraint; MR = mechanical restraint; and SD = standard deviation.

**Table 3:** Studies on KAE regarding R/S among individuals with mental illness.

Reference	Study Design	Participants and Setting	Sampling	Methods	Main Findings		
					Knowledge	Attitude	Experience
Yahyavi & Shahvari [16]	Qualitative, hermeneutic, phenomenological approach	20 patients from an inpatient psychiatric hospital in Tehran, Iran.	Purposive	In-depth unstructured interview	NA	NA	Four main themes and twelve sub-themes Captivity (limitation and suffocation). Abuse (humiliation, violation, and harassment). Oppression (innocence, sadness, hatred, and fear). Punishment (waiting for freedom for reflection and waiting for recovery).
Kamel et al. [33]	Descriptive exploratory design	80 psychotic patients from a psychiatric hospital in Alexandria, Egypt.	Stratified random sampling	Structured interview schedule	NA	NA	The most common feelings experienced are humiliation and worthlessness (38.75%) and rage and resentment (25%), while 3.75% feel restraints make them calm down. Number of times being restrained during hospital stay: Once (52.5%); More than once (47.5%). Physical reaction to restraint (85%): General body aches (100%); Severe pain in extremities (4.4%). Alternatives to restraint: Devoting time and providing care (65%); Giving medication (13.75%); No alternatives (21.25%). Causes of restraint offered by patients and nurses were congruent.
Elgamal [34]	Descriptive correlation, Cross-sectional	30 patients from Kuwait state Psychological Medicine Hospital (4 acute wards were included: 2 male wards and 2 female wards)	Systematic random	One questionnaire	NA	46% reported that restraint is not accepted. 86% reported feeling helpless. 66% said that aggression is the result of PR. 63% reported feeling as if they were being punished. 90% reported experiencing mixed, confusing emotions.	NA

NR = not reported; NA = not applicable; R/S = restraint/seclusion; PR = physical restraint; MR = mechanical restraint; and SD = standard deviation.

**Table 4:** Summary of the appraisal of the methodological quality of the studies included in this review (JBI Critical Appraisal Tools).

Reference	Study Design	* JBI Tool	Total Items (n)	Items ‘Yes’ (n)	Score (%)	Quality Rating
Aslam et al. [18]	Cross-sectional	CS	8	7	87.5	High
Thomas [21]	Cross-sectional	CS	8	7	87.5	High
Abdel-Hussein & Mohamed [25]	Cross-sectional	CS	8	5	62.5	Moderate
Ahmed et al. [24]	Cross-sectional	CS	8	6	75.0	High
Abdulla et al. [19]	Cross-sectional	CS	8	7	87.5	High
Maatouk et al. [20]	Cross-sectional	CS	8	6	75.0	High
Aloulou et al., [23]	Cross-sectional	CS	8	4	50.0	Moderate
Hasan & Abulattifah [27]	Cross-sectional	CS	8	7	87.5	High
Khalil et al. [28]	Cross-sectional	CS	8	6	75.0	High
Mahmoud [29]	Cross-sectional	CS	8	6	75.0	High
Al-Awawdeh et al. [30]	Cross-sectional	CS	8	6	75.0	High
Dawood [31]	Cross-sectional	CS	8	4	50.0	Moderate
Morsi & Sabra [32]	Cross-sectional	CS	8	5	62.5	Moderate
Yahyavi & Shahvari [16]	Qualitative	Qual	10	8	80.0	High
Moghadam et al. [17]	Qualitative	Qual	10	4	40.0	Low
Elgamal [34]	Observational	Prev	9	3	33.3	Low
Kamel et al. [33]	Observational	Prev	9	5	55.6	Moderate
El Kefi et al. [22]	Observational	Prev	9	6	66.7	Moderate
Al-Maraira et al. [26]	Observational	Prev	9	7	77.8	High

* JBI designates the Joanna Briggs Institute; CS is represented by the Analytical Cross-Sectional Studies checklist (8 items); Qual denotes a checklist for Qualitative Research (10 items); and Prev refers to a checklist used to evaluate Prevalence Studies (9 items). The definitions that have been adopted to classify quality ratings are as follows: high = (≥75% of applicable items rated “Yes”); moderate = (50–74%); low = (<50%). The quality ratings were established based upon independent, dual-reviewer assessment with any disagreements resolved through discussion or by reference to a third reviewer where necessary (O.P., K.L.).

## Data Availability

The original contributions presented in this study are included in the article/Supplementary Materials. Further inquiries can be directed to the corresponding author.

## Supplementary Materials

The following supporting information can be downloaded at: https://www.mdpi.com/article/10.3390/healthcare14091161/s1, File S1: Database-Specific Search Strings; File S2: Backward and Forward Citation Searching Results; File S3: Adaptation of the CROSS Checklist for Non-Survey Study Designs; Table S1: PRISMA 2020 Checklist; Table S2: PRISMA 2020 for Abstracts Checklist. Reference [12] is cited in the Supplementary Materials.
