# Supplementary material for: Physical Restraints and Seclusion in Psychiatric Settings in the Eastern Mediterranean Region: A Systematic Review of the Perspectives of Nurses and Individuals with Mental Illness
Source: Healthcare (Basel). 2026 Apr 26;14(9):1161. doi: 10.3390/healthcare14091161 (PMC13163978; doi:10.3390/healthcare14091161)
Supplement: Supplementary file 1 [file healthcare-14-01161-s001.zip › healthcare-4161467-supplementary(1).pdf]

File S1

Database-Specific Search Strings

Overview

Two separate searches were executed per database:

- **Search 1** targeted the perspectives of **nursing staff**, combining the mental health context block, nursing staff block, restraint/seclusion block, cognition and affect block, and geographic location block.
- **Search 2** targeted the perspectives of **individuals with mental illness**, substituting the nursing staff block with a service user block; all other blocks remained identical.

Search results from all databases were exported to Covidence (Veritas Health Innovation) for deduplication and screening.

Conceptual Block Structure

Block	Label	Terms
Block 1	Mental Health Context	mental health, psychiatr*, mental illness, mental disorder*, mental health problem*, mental health service*
Block 2	Nursing Staff	mental health nurs*, psychiatric nurs*, nurs*, nursing staff, mental health professional*, healthcare professional*
Block 3	Service Users	psychiatric patient*, mentally ill, people with mental illness, individuals with mental illness, psychotic patient*, mental health service user*
Block 4	Restraint and Seclusion	restraint*, physical restraint*, mechanical restraint*, manual restraint*, seclusion, secluded, isolation room, isolation, coercive measure*, coercive strateg*, therapeutic restraint*, containment
Block 5	Cognition and Affect	knowledge, attitude*, perception*, experience*, practice*, belief*, values, behaviour*, behavior*, thinking, thought, feeling*
Block 6	Geographic Location	Eastern Mediterranean, Eastern Mediterranean Region, Afghanistan, Bahrain, Djibouti, Egypt, Iran, Iraq, Jordan, Kuwait, Lebanon, Libya, Morocco, Occupied Palestinian territory, Oman, Pakistan, Palestine,

		Qatar, Saudi Arabia, Somalia, Sudan, Syria, Tunisia, United Arab Emirates, UAE, Yemen

Search 1 = Blocks 1 AND 2 AND 4 AND 5 AND 6

Search 2 = Blocks 1 AND 3 AND 4 AND 5 AND 6

A1. PubMed

Search 1 - Nursing Staff Perspectives

(("mental health"[tiab] OR "psychiatr*"[tiab] OR "mental illness"[tiab]
OR "mental disorder*"[tiab] OR "mental health service*"[tiab]))
AND
(("nurs*"[tiab] OR "psychiatric nurs*"[tiab] OR "mental health nurs*"[tiab]
OR "nursing staff"[tiab] OR "healthcare professional*"[tiab])
OR "Nurses"[MeSH] OR "Psychiatric Nursing"[MeSH])
AND
(("restraint*"[tiab] OR "physical restraint*"[tiab] OR "mechanical restraint*"[tiab]
OR "manual restraint*"[tiab] OR "seclusion"[tiab] OR "isolation room"[tiab]
OR "coercive measure*"[tiab] OR "therapeutic restraint*"[tiab] OR "containment"[tiab])
OR "Restraint, Physical"[MeSH])
AND
(("knowledge"[tiab] OR "attitude*"[tiab] OR "perception*"[tiab] OR "experience*"[tiab]
OR "practice*"[tiab] OR "belief*"[tiab] OR "behaviour*"[tiab] OR "behavior*"[tiab])
OR "Health Knowledge, Attitudes, Practice"[MeSH])
AND
("Eastern Mediterranean"[tiab] OR "Afghanistan"[tiab] OR "Bahrain"[tiab]
OR "Egypt"[tiab] OR "Iran"[tiab] OR "Iraq"[tiab] OR "Jordan"[tiab]
OR "Kuwait"[tiab] OR "Lebanon"[tiab] OR "Libya"[tiab] OR "Morocco"[tiab]
OR "Pakistan"[tiab] OR "Palestine"[tiab] OR "Qatar"[tiab] OR "Saudi Arabia"[tiab]
OR "Sudan"[tiab] OR "Syria"[tiab] OR "Tunisia"[tiab] OR "United Arab Emirates"[tiab]
OR "Yemen"[tiab] OR "Oman"[tiab] OR "Djibouti"[tiab] OR "Somalia"[tiab])

Filters applied: English or Arabic; Humans

Search 2 - Perspectives of Individuals with Mental Illness

Block 2 (nursing staff) is replaced with the following service user block. All other blocks remain identical to Search 1.

```
("psychiatric patient*"[tiab] OR "mentally ill"[tiab]
OR "people with mental illness"[tiab] OR "individuals with mental illness"[tiab]
OR "mental health service user*"[tiab] OR "psychotic patient*"[tiab]
OR "Mental Disorders"[MeSH])
```

A2. MEDLINE via Ovid

Search 1 - Nursing Staff Perspectives

```
1. (mental health OR psychiatr* OR mental illness OR mental disorder*
   OR mental health service*).mp.
2. (nurs* OR psychiatric nurs* OR mental health nurs* OR nursing staff
   OR healthcare professional*).mp.
3. (restraint* OR physical restraint* OR mechanical restraint* OR manual restraint*
   OR seclusion OR secluded OR isolation room OR coercive measure*
   OR therapeutic restraint* OR containment).mp.
4. (knowledge OR attitude* OR perception* OR experience* OR practice*
   OR belief* OR behaviour* OR behavior* OR feeling*).mp.
5. (Eastern Mediterranean OR Afghanistan OR Bahrain OR Djibouti OR Egypt OR Iran
   OR Iraq OR Jordan OR Kuwait OR Lebanon OR Libya OR Morocco OR Oman
   OR Pakistan OR Palestine OR Qatar OR Saudi Arabia OR Somalia OR Sudan
   OR Syria OR Tunisia OR United Arab Emirates OR UAE OR Yemen).mp.
6. 1 AND 2 AND 3 AND 4 AND 5

Limits: English or Arabic; Humans
```

Search 2 - Perspectives of Individuals with Mental Illness

Replace line 2 with the following; all other lines remain identical.

```
2. (psychiatric patient* OR mentally ill OR people with mental illness
   OR individuals with mental illness OR mental health service user*
   OR psychotic patient*).mp.
```

A3. EMBASE via Ovid

Search 1 - Nursing Staff Perspectives

1. (mental health OR psychiatr* OR mental illness OR mental disorder*
   OR mental health service*).mp.
2. (nurs* OR psychiatric nurs* OR mental health nurs* OR nursing staff
   OR healthcare professional*).mp.
3. (restraint* OR physical restraint* OR mechanical restraint* OR manual restraint*
   OR seclusion OR secluded OR isolation OR coercive measure*
   OR coercive strateg* OR therapeutic restraint* OR containment).mp.
4. (knowledge OR attitude* OR perception* OR experience* OR practice*
   OR belief* OR behaviour* OR behavior* OR feeling*).mp.
5. (Eastern Mediterranean OR Afghanistan OR Bahrain OR Djibouti OR Egypt OR Iran
   OR Iraq OR Jordan OR Kuwait OR Lebanon OR Libya OR Morocco OR Oman
   OR Pakistan OR Palestine OR Qatar OR 'Saudi Arabia' OR Somalia OR Sudan
   OR Syria OR Tunisia OR 'United Arab Emirates' OR UAE OR Yemen).mp.
6. 1 AND 2 AND 3 AND 4 AND 5

Limits: English or Arabic; Humans

Search 2 -Perspectives of Individuals with Mental Illness

Replace line 2 with the following; all other lines remain identical.

2. (psychiatric patient* OR mentally ill OR people with mental illness
   OR individuals with mental illness OR mental health service user*
   OR psychotic patient*).mp.

A4. CINAHL (EBSCOhost)

Search 1 - Nursing Staff Perspectives

S1: TI,AB (mental health OR psychiatr* OR mental illness OR mental disorder*
OR mental health service*)
S2: TI,AB (nurs* OR "psychiatric nurs*" OR "mental health nurs*" OR "nursing staff"
OR "healthcare professional*")

OR MH "Nurses" OR MH "Psychiatric Nursing"
S3: TI,AB (restraint* OR "physical restraint*" OR "mechanical restraint*"
OR "manual restraint*" OR seclusion OR secluded OR "isolation room"
OR "coercive measure*" OR "therapeutic restraint*" OR containment)
OR MH "Restraint, Physical"
S4: TI,AB (knowledge OR attitude* OR perception* OR experience* OR practice*
OR belief* OR behaviour* OR behavior*)
OR MH "Health Knowledge, Attitudes, Practice"
S5: TI,AB ("Eastern Mediterranean" OR Afghanistan OR Bahrain OR Djibouti
OR Egypt OR Iran OR Iraq OR Jordan OR Kuwait OR Lebanon OR Libya
OR Morocco OR Oman OR Pakistan OR Palestine OR Qatar OR "Saudi Arabia"
OR Somalia OR Sudan OR Syria OR Tunisia OR "United Arab Emirates" OR Yemen)
S6: S1 AND S2 AND S3 AND S4 AND S5

Limiters: English or Arabic; Human

Search 2 - Perspectives of Individuals with Mental Illness

Replace S2 with the following; all other sets remain identical.

S2: TI,AB ("psychiatric patient*" OR "mentally ill" OR "people with mental illness"
OR "individuals with mental illness" OR "mental health service user*"
OR "psychotic patient*")

A5. PsycINFO (APA PsycNet)

Search 1 - Nursing Staff Perspectives

(DE "Nurses" OR DE "Psychiatric Nurses"
OR TI,AB (nurs* OR "psychiatric nurs*" OR "mental health nurs*"
OR "healthcare professional*"))
AND
(DE "Physical Restraint" OR DE "Seclusion"
OR TI,AB (restraint* OR "physical restraint*" OR "mechanical restraint*"
OR seclusion OR "coercive measure*" OR isolation OR containment))
AND
(DE "Attitudes" OR DE "Knowledge"

```
OR TI,AB (knowledge OR attitude* OR perception* OR experience* OR practice*
OR belief* OR behaviour* OR behavior*))
AND
TI,AB ("Eastern Mediterranean" OR Afghanistan OR Bahrain OR Djibouti
OR Egypt OR Iran OR Iraq OR Jordan OR Kuwait OR Lebanon OR Libya
OR Morocco OR Oman OR Pakistan OR Palestine OR Qatar OR "Saudi Arabia"
OR Somalia OR Sudan OR Syria OR Tunisia OR "United Arab Emirates" OR Yemen)

Filters: English or Arabic
```

Search 2 - Perspectives of Individuals with Mental Illness

Replace the first block with the following; all other blocks remain identical.

```
(DE "Mental Patients" OR DE "Psychiatric Patients"
OR TI,AB ("psychiatric patient*" OR "mentally ill"
OR "people with mental illness" OR "individuals with mental illness"
OR "mental health service user*" OR "psychotic patient*"))
```

A6. PsycARTICLES (APA PsycNet)

The same search strings as detailed in A5 (PsycINFO) were applied within the PsycARTICLES database using the APA PsycNet interface.

A7. Web of Science (Core Collection)

Search 1 - Nursing Staff Perspectives

```
TS=(mental health OR psychiatr* OR mental illness OR mental disorder*
   OR mental health service*)
AND TS=(nurs* OR "psychiatric nurs*" OR "mental health nurs*" OR "nursing staff"
      OR "healthcare professional*")
AND TS=(restraint* OR "physical restraint*" OR "mechanical restraint*"
      OR "manual restraint*" OR seclusion OR secluded OR "isolation room"
      OR "coercive measure*" OR "therapeutic restraint*" OR containment)
AND TS=(knowledge OR attitude* OR perception* OR experience* OR practice*
```

```
    OR belief* OR behaviour* OR behavior*)
AND TS=("Eastern Mediterranean" OR Afghanistan OR Bahrain OR Djibouti
    OR Egypt OR Iran OR Iraq OR Jordan OR Kuwait OR Lebanon OR Libya
    OR Morocco OR Oman OR Pakistan OR Palestine OR Qatar OR "Saudi Arabia"
    OR Somalia OR Sudan OR Syria OR Tunisia OR "United Arab Emirates" OR Yemen)

Refined by: Languages = English OR Arabic
```

Search 2 - Perspectives of Individuals with Mental Illness

Replace the second TS line with the following; all other lines remain identical.

```
AND TS=("psychiatric patient*" OR "mentally ill" OR "people with mental illness"
    OR "individuals with mental illness" OR "mental health service user*"
    OR "psychotic patient*")
```

A8. ASSIA (ProQuest)

Search 1 - Nursing Staff Perspectives

```
(nurs* OR "psychiatric nurs*" OR "mental health nurs*" OR "healthcare professional*")
AND (restraint* OR "physical restraint*" OR "mechanical restraint*" OR seclusion
  OR "coercive measure*" OR isolation OR containment)
AND (knowledge OR attitude* OR perception* OR experience* OR practice* OR belief*)
AND ("Eastern Mediterranean" OR Afghanistan OR Bahrain OR Djibouti OR Egypt
  OR Iran OR Iraq OR Jordan OR Kuwait OR Lebanon OR Libya OR Morocco OR Oman
  OR Pakistan OR Palestine OR Qatar OR "Saudi Arabia" OR Somalia OR Sudan
  OR Syria OR Tunisia OR "United Arab Emirates" OR Yemen)

Language filter: English OR Arabic
```

Search 2 - Perspectives of Individuals with Mental Illness

Replace the first line with the following; all other lines remain identical.

```
("psychiatric patient*" OR "mentally ill" OR "people with mental illness"
OR "individuals with mental illness" OR "mental health service user*"
OR "psychotic patient*")
```

A9. Google Scholar

Search 1 - Nursing Staff Perspectives

("physical restraint" OR "mechanical restraint" OR seclusion OR "coercive measures")
AND ("mental health nurse*" OR "psychiatric nurse*" OR "nursing staff")
AND (knowledge OR attitudes OR experience* OR perceptions OR practices)
AND ("Eastern Mediterranean" OR "Saudi Arabia" OR Egypt OR Iran OR Iraq
OR Jordan OR Kuwait OR Tunisia OR Sudan OR Palestine OR Pakistan
OR Lebanon OR Libya OR Morocco OR Bahrain OR Oman OR Qatar OR Yemen
OR Syria OR "United Arab Emirates")

Search 2 - Perspectives of Individuals with Mental Illness

("physical restraint" OR "mechanical restraint" OR seclusion OR "coercive measures")
AND ("psychiatric patient*" OR "mentally ill" OR "people with mental illness"
OR "individuals with mental illness")
AND (knowledge OR attitudes OR experience* OR perceptions)
AND ("Eastern Mediterranean" OR "Saudi Arabia" OR Egypt OR Iran OR Iraq
OR Jordan OR Kuwait OR Tunisia OR Sudan OR Palestine OR Pakistan
OR Lebanon OR Libya OR Morocco OR Bahrain OR Oman OR Qatar OR Yemen
OR Syria OR "United Arab Emirates")

Note: The first 20 pages of search results (approximately 200 records per search) were systematically reviewed. All records identified through Google Scholar were exported to Covidence for deduplication and screening alongside records retrieved from the other eight databases.

File S2

Backward and Forward Citation Searching Results

Overview

Backward and forward citation searching was conducted for all 19 studies meeting the final inclusion criteria. Backward citation searching involved reviewing the reference lists of each included article. Forward citation searching was conducted via Google Scholar and Web of Science to identify studies that had subsequently cited each included article. No additional studies meeting the predefined eligibility criteria were identified through either procedure.

Table B1. Backward and Forward Citation Counts for All Included Studies

No.	Study Title	Backward Citations Reviewed (n)	Forward Citations Reviewed (n)	Additional Eligible Studies Identified (n)
1	Knowledge, Attitude and Practices of Psychiatric Nurses towards the Use of Physical Restraints in a tertiary care hospital Lahore, Pakistan	17	2	0
2	Role of nurses and the nursing assistants in the implementation and monitoring of physical restraint in psychiatry	0	0	0
3	Therapeutic isolation in psychiatry	0	0	0
4	Reactions of patients and psychiatric hospital staff about physical restraint	58	13	0
5	Assess nurses' knowledge, attitudes and practice toward physical restraint for psychiatric patients	20	4	0
6	Psychiatric Nurses' Perceptions about Physical Restraint: A Qualitative Study	26	54	0

7	A Descriptive Study to Evaluate Registered Nurses' Knowledge, Attitude and Practice of Physical Restraints in King Salman Armed Forces Hospital in Tabuk, Kingdom of Saudi Arabia in 2019	16	0	0
8	Psychiatric staff attitudes toward coercive measures: An experimental design	34	9	0
9	Nurses' knowledge, attitudes, and practices toward physical restraint and seclusion in an inpatients' psychiatric ward	97	25	0
10	Psychiatric nurses' knowledge, attitudes, and practice towards the use of physical restraints	20	18	0
11	Nurses' Attitudes and Practices towards Inpatient Aggression in a Palestinian Mental Health Hospital	42	3	0
12	The Current Knowledge and Attitude of Nurses Working at Tanta Mental Health Hospital Regarding The Use of Physical Restraint on Psychiatric Patients	25	0	0
13	Beliefs and Attitudes of Health Care Professionals Toward Mental Health Services Users' Rights: A Cross-Sectional Study from the United Arab Emirates	34	2	0
14	Patients and staff attitudes toward physical restraint	27	8	0
15	Evaluation of nursing knowledge on physical restraint practiced in psychiatry	0	0	0
16	Psychiatric Nurses' Attitude and Practice toward Physical Restraint	45	52	0
17	Psychiatric Inpatients' Lived Experiences of Physical Restraint: A Qualitative Study in Iran	20	3	0
18	Assessment of nurses' knowledge about restraint and seclusion used for aggressive patients in psychiatric wards	15	0	0
19	Causes and management of psychiatric inpatient aggression and violence: Comparison between Egyptian and Saudi nurses' perspectives	43	10	0
	**Total**	**539**	**203**	**0**

Note: Backward citation searching was performed by manually reviewing the reference list of each included article. Forward citation searching was conducted using Google Scholar and Web of Science. All records identified were screened against the predefined eligibility criteria by the lead reviewer (A.S.A.). No additional eligible studies were identified through either procedure.

File S3

Adaptation of the CROSS Checklist for Non-Survey Study Designs

Background

The CROSS (Consensus-Based Checklist for Reporting of Survey Studies) checklist was originally developed by Sharma et al. (2021) to assess the completeness and transparency of reporting in survey-based research. As the present review included studies using a range of designs — cross-sectional questionnaire surveys (n = 15), qualitative interviews (n = 2), an observational checklist study (n = 1), and an assessment-structured interview schedule study (n = 1) — several CROSS items required adaptation to remain applicable and meaningful across this methodological diversity.

All adaptations were agreed upon by two independent reviewers (A.S.A. and A.H.A.) prior to commencing the quality assessment process. The adapted checklist was applied uniformly to all 19 included studies, with items scored as Met / Not Met / Unclear.

Scoring Key

- **Retained:** Item applied as originally worded in the CROSS checklist.
- **Reworded:** Item adapted in wording to be applicable across survey and non-survey designs; the underlying construct being assessed remained unchanged.
- **Omitted (non-survey only):** Item excluded from scoring for qualitative and observational studies, as it is not applicable to those designs. Item was retained and scored for survey-based studies.

Table C1. CROSS Checklist Adaptation for Non-Survey Study Designs

Item No.	Original CROSS Item (Sharma et al., 2021)	Section	Adaptation Status	Adapted Wording (if reworded)	Rationale for Adaptation or Omission
1	Title identifies the study as a survey	Title	**Omitted (non-survey only)**	—	Not applicable to qualitative interview or observational studies, which are not surveys
2	Abstract includes structured information: background,	Abstract	**Retained**	—	Applicable to all study designs included in this review

	objectives, methods, results, and conclusions				
3	Scientific background and rationale for the study are provided	Introduction	**Retained**	—	Applicable to all study designs
4	Specific objectives or hypotheses are clearly stated	Introduction	**Retained**	—	Applicable to all study designs
5	Study design is explicitly stated	Methods	**Retained**	—	Applicable to all study designs
6	Ethical approval and participant informed consent are described	Methods	**Retained**	—	Applicable to all study designs
7	Target population and eligibility criteria are clearly defined	Methods	**Retained**	—	Applicable to all study designs
8	Sampling frame and sampling technique are described	Methods	**Reworded**	"Participant selection approach and recruitment procedure are described"	The term "sampling frame" is specific to survey methodology; the adapted wording captures the same construct across qualitative and observational designs
9	Sample size calculation or justification is provided	Methods	**Reworded**	"Rationale for sample size is provided, or data saturation is described (for qualitative studies)"	Power calculations are not applicable to qualitative research; saturation is the qualitative equivalent
10	Survey tool is described and cited or provided	Methods	**Reworded**	"Data collection instrument or procedure is described in sufficient detail"	Broadened to encompass interview guides, observational checklists, and assessment schedules in addition to questionnaires
11	Pilot testing of the survey instrument is reported	Methods	**Omitted (non-survey only)**	—	Pilot testing of a formal instrument is not applicable to

					unstructured or semi-structured qualitative interviews or observational checklists
12	Validity and reliability of the survey instrument are described	Methods	**Reworded**	"Rigour or trustworthiness measures are described (e.g., inter-rater reliability for quantitative studies; reflexivity, member checking, or triangulation for qualitative studies)"	Validity and reliability as defined in survey research have qualitative equivalents that must be acknowledged
13	Survey administration and data collection process are described	Methods	**Reworded**	"Data collection process, setting, and timeframe are clearly described"	Adapted to encompass interview and observational data collection procedures
14	Response rate is reported	Results	**Omitted (non-survey only)**	—	Response rate is a survey-specific metric; not applicable to interview-based or observational studies
15	Non-response analysis or comparison with non-respondents is conducted	Results	**Omitted (non-survey only)**	—	Not applicable to non-survey designs; non-response bias is a survey-specific concern
16	Statistical methods are described	Methods	**Reworded**	"Data analysis method is described (statistical methods for	Broadened to include qualitative analysis approaches

				quantitative studies; thematic or content analysis for qualitative studies)"	
17	Demographic and clinical characteristics of participants are reported	Results	**Retained**	—	Applicable to all study designs
18	Missing data are reported and handled appropriately	Results	**Retained**	—	Applicable to all study designs
19	Findings are clearly presented for each stated objective	Results	**Retained**	—	Applicable to all study designs
20	Subgroup analyses are described where applicable	Results	**Reworded**	"Variation in findings across subgroups or themes is described where applicable"	Adapted to include thematic differentiation in qualitative findings
21	Key findings are discussed in the context of existing evidence	Discussion	**Retained**	—	Applicable to all study designs
22	Study limitations are acknowledged	Discussion	**Retained**	—	Applicable to all study designs
23	Generalisability or transferability of findings is discussed	Discussion	**Reworded**	"Generalisability (quantitative) or transferability (qualitative) of findings is discussed"	Transferability is the qualitative equivalent of generalisability
24	Conclusions are supported by the study data	Discussion	**Retained**	—	Applicable to all study designs

Summary of Adaptations

Adaptation Status	Number of Items	Item Numbers
Retained without modification	13	2, 3, 4, 5, 6, 7, 17, 18, 19, 21, 22, 24

Reworded for cross-design applicability	7	8, 9, 10, 12, 13, 16, 20, 23
Omitted for non-survey designs	4	1, 11, 14, 15
**Total CROSS items**	**24**	

Note: Items 1, 11, 14, and 15 were retained in full for the 15 survey-based studies included in this review and scored accordingly. They were omitted only when applied to the two qualitative interview studies, one observational checklist study, and one assessment-structured interview schedule study.
