# Supplementary material for: Physical Restraints and Seclusion in Psychiatric Settings in the Eastern Mediterranean Region: A Systematic Review of the Perspectives of Nurses and Individuals with Mental Illness
Source: Healthcare (Basel). 2026 Apr 26;14(9):1161. doi: 10.3390/healthcare14091161 (PMC13163978; doi:10.3390/healthcare14091161)
Supplement: Supplementary file 1 [file healthcare-14-01161-s001.zip › PRISMA 2020 Checklist.pdf]

PRISMA 2020 Checklist (Table S1)

Physical Restraints and Seclusion in Psychiatric Settings in the Eastern Mediterranean Region: A Systematic Review of the Perspectives of Nurses and Individuals with Mental Illness

Section	Item	Checklist Item Description	Location in Manuscript
**TITLE**	1	Identify the report as a systematic review.	Title page, p. 1
**ABSTRACT**	2	See the PRISMA 2020 for Abstracts checklist (Table 2).	Abstract, p. 1
**INTRODUCTION Rationale**	3	Describe the rationale for the review in the context of existing knowledge.	Section 1 (Introduction), pp. 2–3
**INTRODUCTION Objectives**	4	Provide an explicit statement of the objective(s) or question(s) the review addresses.	Section 1 (Introduction), p. 3
**METHODS Eligibility criteria**	5	Specify the inclusion and exclusion criteria for the review and how studies were grouped for the syntheses.	Section 2.1, pp. 3–4
**METHODS Information sources**	6	Specify all databases, registers, websites, organisations, reference lists and other sources searched or consulted to identify studies. Specify the date when each source was last searched or consulted.	Section 2.3, p. 4
**METHODS Search strategy**	7	Present the full search strategies for all databases, registers and websites, including any filters and limits used.	Section 2.3, p. 4–5; Supplementary Appendix A
**METHODS Selection process**	8	Specify the methods used to decide whether a study met the inclusion criteria of the review, including how many reviewers screened each record and each report retrieved, whether they worked independently, and if applicable, details of automation tools used in the process.	Section 2.4, pp. 5–6
**METHODS Data collection process**	9	Specify the methods used to collect data from reports, including how many reviewers collected data from each report, whether they worked independently, and any processes for obtaining or confirming data from study investigators.	Section 2.5, pp. 6–7
**METHODS Data items**	10	List and define all outcomes for which data were sought. Specify whether all results that were compatible with each outcome domain in each study were sought, and if not, the methods used to decide which results to collect.	Section 2.5, pp. 6–7
**METHODS Study risk of bias assessment**	11	Specify the methods used to assess risk of bias in the included studies, including details of the tool(s) used, how many reviewers assessed each study and whether they worked independently.	Section 2.6, pp. 7–8

**METHODS**<br>**Effect measures**	12	Specify for each outcome the effect measure(s) used in the synthesis or presentation of results.	Not applicable (narrative synthesis)
**METHODS**<br>**Synthesis methods**	13	Describe the processes used to decide which studies were eligible for each synthesis, how studies were prepared for synthesis, and any processes used to identify an appropriate statistical model or method.	Section 2.7, pp. 8–9
**METHODS**<br>**Reporting bias assessment**	14	Describe any methods used to assess risk of bias due to missing results in a synthesis (arising from reporting bias).	Section 2.6, p. 7–8;<br>Section 2.7, p. 8
**METHODS**<br>**Certainty assessment**	15	Describe any methods used to assess certainty (or confidence) in the body of evidence for an outcome.	Section 2.6, pp. 7–8
**RESULTS**<br>**Study selection**	16	Describe the results of the search and selection process, including the number of records identified, screened, assessed for eligibility, and included, with reasons for exclusions.	Section 3.1, pp. 9–10;<br>Figure 1 (PRISMA flow diagram)
**RESULTS**<br>**Study characteristics**	17	Cite each included study and present its characteristics.	Section 3.1, pp. 9–10;<br>Table 1; Tables 2–3
**RESULTS**<br>**Risk of bias in studies**	18	Present assessments of risk of bias (methodological quality) for each included study.	Section 3.5, pp. 16–17;<br>Table 4
**RESULTS**<br>**Results of individual studies**	19	For all outcomes, present summary statistics and effect estimates for each study, ideally in a structured table or figure.	Sections 3.2–3.3, pp. 10–15;<br>Tables 2–3
**RESULTS**<br>**Results of syntheses**	20	For each synthesis, briefly summarise the characteristics and risk of bias among contributing studies and present results of all statistical syntheses conducted.	Sections 3.2–3.3, pp. 10–15;<br>Section 3.6, p. 17
**RESULTS**<br>**Reporting biases**	21	Present assessments of risk of bias due to missing results (arising from reporting bias) for each synthesis assessed.	Section 3.4, pp. 15–16;<br>Supplementary Appendix C
**RESULTS**<br>**Certainty of evidence**	22	Present assessments of certainty (or confidence) in the body of evidence for each outcome assessed.	Section 4.6 (Limitations), pp. 22–23
**DISCUSSION**<br>**Discussion**	23	Provide a general interpretation of the results in the context of other evidence.	Section 4, pp. 18–23
**DISCUSSION**<br>**Limitations**	24	Discuss any limitations of the evidence included in the review, and limitations of the review process used.	Section 4.6, pp. 22–23
**DISCUSSION**<br>**Conclusions**	25	Provide a general interpretation of the results in the context of other evidence, and implications for future research.	Section 5 (Conclusions), p. 23–24;<br>Abstract, p. 1
**OTHER INFORMATION**<br>**Registration and protocol**	26	Provide registration information for the review, including register name and registration number, or state that the review was not registered. Indicate where the review protocol can be accessed, or state that a protocol was not prepared.	Section 2.2, p. 4 (PROSPERO: CRD42023383751)
**OTHER**	27	Describe sources of financial or other	Funding section, p. 24

**INFORMATION Support**		support for the review, and the role of the funders or sponsors in the review.	
**OTHER INFORMATION Competing interests**	28	Declare any competing interests of review authors.	Conflicts of Interest section, p. 24
**OTHER INFORMATION Availability of data, code and other materials**	29	Report which of the following are publicly available and where they can be found: template data collection forms; data extracted from included studies; data used for all analyses; analytic code; any other materials used in the review.	Data Availability Statement, p. 24; Supplementary Appendices A–C

Note: This checklist was completed in accordance with Page et al. (2021) — PRISMA 2020 statement: an updated guideline for reporting systematic reviews. BMJ, 372, n71. https://doi.org/10.1136/bmj.n71. Items marked "Not applicable" reflect the narrative synthesis design of this review, which did not employ meta-analytic statistical pooling.

PRISMA 2020 for Abstracts Checklist (Table S2)

Section	Item	Checklist Item Description	Location in Manuscript
**TITLE**	A1	Identify the report as a systematic review.	Title page, p. 1
**BACKGROUND**	A2	Provide a brief background of the review topic.	Abstract (Background/Objectives), p. 1
**OBJECTIVES**	A3	Provide an explicit statement of the objective(s) or question(s) the review addresses.	Abstract (Background/Objectives), p. 1
**ELIGIBILITY CRITERIA**	A4	Specify the inclusion and exclusion criteria.	Abstract (Methods), p. 1
**INFORMATION SOURCES**	A5	Specify the information sources used to identify studies and the date when each was last searched.	Abstract (Methods), p. 1
**RISK OF BIAS**	A6	Specify the methods used to assess risk of bias in the included studies.	Abstract (Methods), p. 1
**SYNTHESIS OF RESULTS**	A7	Specify the methods used to present and synthesise results.	Abstract (Methods), p. 1
**INCLUDED STUDIES**	A8	Give the total number of included studies and participants and summarise relevant characteristics of studies.	Abstract (Results), p. 1
**SYNTHESIS OF RESULTS**	A9	Present the main results, including, for each outcome, the number of contributing studies and participants.	Abstract (Results), p. 1
**LIMITATIONS OF EVIDENCE**	A10	Provide a brief summary of the limitations of the evidence included in the review.	Abstract (Conclusions), p. 1
**INTERPRETATION**	A11	Provide a general interpretation of the results and important implications.	Abstract (Conclusions), p. 1
**FUNDING**	A12	Specify the primary source of funding for the review.	Abstract / Funding, p. 1 / p. 24
**REGISTRATION**	A13	Provide the register name and registration number.	Abstract (Methods), p. 1 (PROSPERO: CRD42023383751)

Note: This checklist was completed in accordance with Page et al. (2021) — PRISMA 2020 abstract checklist. BMJ, 372, n71. https://doi.org/10.1136/bmj.n71
